# Anti-inflammatory and Antioxidant Properties of Probiotic Bacterium *Lactobacillus mucosae* AN1 and *Lactobacillus fermentum* SNR1 in Wistar Albino Rats

**DOI:** 10.3389/fmicb.2018.03063

**Published:** 2018-12-14

**Authors:** Repally Ayyanna, Dasari Ankaiah, Venkatesan Arul

**Affiliations:** Department of Biotechnology, School of Life Sciences, Pondicherry University, Pondicherry, India

**Keywords:** microencapsulation, anti-inflammation, wistar rats, interleukins, histopathology, immunohisto chemistry, qRT-PCR, antioxidant activity

## Abstract

The potent antioxidant probiotic strains *Lactobacillus mucosae* AN1 and *Lactobacillus fermentum* SNR1 were assessed for anti-inflammatory properties in carrageenan (acute) and complete Freund’s adjuvant-induced inflammation (chronic) models in the present study. The two probiotic strains were administered orally along with feed to the Wistar albino male rats as whole cell as well as microencapsulated form. The following experiments were performed to evaluate the anti-inflammatory properties of probiotic strains and the results were observed that the encapsulated and unencapsulated probiotic strains have exhibited statistically significant decrease in paw thickness. Percentage of inhibition in paw thickness of microencapsulated probiotic bacteria (Group VIII), unencapsulated strains (Group IX) were revealed 85 ± 13% and 77 ± 25%, respectively. In Hematoxylin and Eosin staining, results were revealed that the probiotic strains were exhibited anti-inflammatory effects on inflammation-induced paw tissues. qRT-PCR studies revealed upregulation of anti-inflammatory cytokine genes and down-regulation pro-inflammatory cytokine genes in probiotic-treated rat paw tissues. Further, the expression of anti-inflammatory and pro-inflammatory cytokines were examined using immunohistochemistry and ELISA methods. The probiotic administered rat paw tissue in different groups have exhibited the low level of lipid peroxides formation and higher anti-oxidant activities when compared to the control and inflammation control tissues.

## Introduction

Lactic bacteria are the major probiotic bacteria that included in functional foods since they are considered as generally regarded as safe status and various benefits to the host organisms. However, probiotics should be able to survive in the host’s gastrointestinal tract (Sahadeva et al., 2011). FAO defined probiotics as “Live microbiota which are administered in sufficient amounts confer a beneficial to the host” (Hill et al., 2014). Probiotics beneficial effects such as antimicrobial, immunomodulatory, anti-cancerous, anti-arthritic properties, production of various vitamins and prevention of gut infections (Salminen et al., 2005). Probiotics in the management of allergic diseases by stabilizing the microbes such as intestine, increase in degradation of antigens, immunogenicity and enhance intestinal health by inhibition of disease causing pathogens (Brady et al., 2000; Marteau et al., 2001).

The immune system detects the inflammation process and creates protein chain circulating immune complex it targets the inflammation conditions (Baniyash, 2004). Inflammation associated symptoms such as tissue injury, granuloma formation and leukocyte infiltration (Medzhitov, 2008). Sixty to eighty percent of the world population relies on the alternative medicine (Bodeker and Kronenberg, 2002). *Lactobacillus mucosae* is a Gram-positive, rod-shaped species, an obligate anaerobe, catalase-negative, non-motile and it has mucus-binding activity. *Lactobacillus mucosae* is a probiotic bacteria was first identified using a gene probe designed for the mucus-binding gene and it was significantly enhanced the adherent ability of *L. reuteri* strains to mucin (Roos et al., 2000; Lee et al., 2012). Probiotic genes identified in *L. mucosae* such as beta-galactosidase and bile salt hydrolase lowers the cholesterol and ameliorates lactose intolerance in the host organism these characteristics indicated its probiotic potential (de Vrese et al., 2001; Chae et al., 2013). *Lactobacillus fermentum* found in human gut microbiota, able to adhere epithelial cells in small intestine and prevents the adhesion of bacterial pathogens (Henriksson et al., 1991; Heinemann et al., 2000; Reid et al., 2001).

Microencapsulation technology has been successfully applied for the encapsulation of probiotic bacteria to protect from gut physiological conditions (Zuidam and Shimoni, 2010). Microencapsulation defined as the technology of packaging materials in small capsules that release contents over prolonged duration of time. Microencapsulation technology has significant interest in the pharmaceutical industry for drug or vaccine delivery and in food industry (Champagne et al., 2005). Sodium alginate microencapsulated probiotic cultures improved resilience to gastric acid, lysozyme and bile salts conditions in the digestive system (Chandramouli et al., 2004). Microencapsulation enhances the viable bacteria and facilitate the controlled delivery in the gastrointestinal tract (Champagne and Fustier, 2007).

Nowadays conventional drug treatments are very limited in their efficiency and safety to control the inflammatory diseases. Therefore, this study was conducted to explore the possibility of anti-inflammatory properties of sodium alginate microencapsulated *L. mucosae* AN1 and *L. fermentum* SNR1 by carrageenan-induced acute inflammation in Wistar albino male rat model.

## Materials and Methods

Wistar albino male rats were procured from the JIPMER, Puducherry. Wistar rats were housed in stainless steel cages (3 rats/cage) at 25°C temperature, 50% relative humidity standard conditions were maintained. After 2 weeks of acclimatization period, rats divided into nine groups of six animals in each group and experimental duration for 40 days. During experimental period body weights and daily feed intake were recorded. The present study was carried out according to the principles and guidelines of animal care (Reg. No. 1159/c/07/CPCSEA). All the protocols used in this study was approved by the Animal Ethics Committee, Pondicherry University (Ref. No. PU/SLS/AH/IAEC/2016/08).

### Probiotic Bacteria, Chemicals

Probiotic bacteria *Lactobacillus mucosae* (GenBank, KM068788), *Lactobacillus fermentum* (GenBank, KM068787) were isolated from sheep milk and fermented rice gruel, respectively (Repally et al., 2017). These probiotic strains were used for the treatment of inflammation induced in hind rats paw tissues. De Man Rogosa and Sharpe media (HiMedia, India) was used for the cultivation of the probiotic strains and they were microencapsulated with sodium alginate (SRL Chemicals, India). Diclofenac sodium (Sigma) administered as a standard anti-inflammatory reference drug, Carrageenan (HiMedia) and Complete Freund’s Adjuvant (Bangalore Genei, India) chemicals were used as inflammation inducing agents in this study.

### Animals and Design of Experiment

During the acclimatization period, all the rats were fed with normal rat feed and nine groups were divided. Each group composed of six rats such as Group I (Control); Group II (Inflammation control) Group III (Diclofenac sodium treated). The probiotic bacteria was administered to the rats in the following groups, Group IV (Unencapsulated *L. mucosae*), Group V (Encapsulated *L. mucosae*), Group VI (Unencapsulated *L. fermentum*), Group VII (Encapsulated *L. fermentum*), Group VIII (Encapsulated *L. mucosae* and *L. fermentum*), and Group IX (Unencapsulated *L. mucosae* and *L. fermentum*). Animals were administered with the specified probiotic strain combinations consisting, 1 mL of culture 10^8^ CFU/ mL/day/oral and microencapsulated probiotic strains were administered for the period of 40 days. Then animal organs such as paw tissues and blood were collected for the biochemical estimation.

### Preparation and Microencapsulation of Probiotic Strains

Probiotic strains were inoculated into sterilized MRS media with L-cysteine hydrochloride and incubated at 37°C for 24 h. *L. mucosae* AN1 and *L. fermentum* SNR1 cultures were suspended with 3% (w/v) sodium alginate and gently mixed for 20 min to obtain the suspension. It was injected through the fine gauge syringes into sterile calcium chloride with sodium chloride. After formation of the beads, recovered by filtration, washed with sterile water, air dried and stored at 4°C in aseptic conditions for administration to the specified rat groups (Solanki et al., 2015).

### Carrageenan Induced Acute Inflammation in Rat Paw Tissues

Carrageenan-induced rat paw edema was induced by subplantar injection of 1% carrageenan in sterile saline into the right hind paws of all the groups except control group (Table 1). The acute inflammation induced in hind rat paws by carrageenan was executed as; rat paw thickness was measured before 20 min of carrageenan injection and after carrageenan injection thickness of rat paw was measured at different time intervals (0, 1, 2, 4, and 24 h) by Plethysmometer to examine the inflammation process. The percentage inhibition of paw thickness was calculated using the formula (Solanki et al., 2015).

**Table 1:** Experimental grouping of Wistar albino rats.

Groups	Group composition	No. of animals
I	Control	6
II	Inflammation control	6
III	Diclofenac sodium (reference drug)	6
IV	Unencapsulated *Lactobacillus mucosae* AN1 (administered group)	6
V	Encapsulated *L. mucosae* (administered group)	6
VI	Unencapsulated *Lactobacillus fermentum* SNR1	6
VII	Encapsulated *L. fermentum* SNR1	6
VIII	Encapsulated *L. mucosae* AN1 and *L. fermentum* SNR1	6
IX	Unencapsulated *L. mucosae* AN1 and *L. fermentum* SNR1	6
	Total number of animals	54

$$\begin{array}{lll} \%\;\mathrm{of inhibition} & = & ({\text{V}}_{\text{t}}-{\text{V}}_{0})\text{ Control}-({\text{V}}_{\text{t}}-{\text{V}}_{0})\text{ Treated}/\\ & & ({\text{V}}_{\text{t}}-{\text{V}}_{0})\text{ Control}\times 100. \end{array}$$

### Hematoxylin and Eosin Staining of Paw Tissue Sections

From all the rats, paw tissue sections were prepared using Microtome Blades, deparaffinized using mild heat and subjected to xylene wash. Then tissue sections were hydrated by decreasing the concentration of absolute ethanol (100–60%) and stained with hematoxylin for 3–5 min, then washed in water for 5 min. After washing, tissue sections were differentiated in 1% acid alcohol (1% HCl in 70% ethanol) for 5 min and washed again in water until the sections were blue in color by ammonia solution. The tissue sections were rinsed with water and stained using 1% Eosin for 15 min and followed by water wash for 5 min then dehydrated with increasing concentration of ethanol. Tissue sections were cleared by xylene, mounted with DPX then observed under the light microscope (20×) (Kingston et al., 2007).

### Hematological Parameters

Blood samples were collected from all the rat groups then WBC, RBC cell counts, hemoglobin content and erythrocyte sedimentation rate (ESR) were estimated, C-reactive protein (CRP) levels were analyzed using ELISA method (Valentini et al., 2015).

### Cytokines Assay for Rat Serum

Serum samples were prepared from the rat blood of grouped animals to determine the IL-10 (anti-inflammatory cytokine) and IL-6, TNF-*α* (proinflammatory cytokines) in pg/mL by ELISA reader (SpectraMax M2^e^). IL-6, TNF-α, and IL-10 ELISA kits were procured from Ray Biotech, United States and experiments were performed according to the manufacturer’s instructions and recommendations (Amdekar et al., 2012).

### Immunohistochemistry of Rat Paw Tissues

Determination of the protective effects of microencapsulated probiotic strains and inflammation in the paw tissues were assessed using immunohistochemistry. Rat paw tissue sections were coated with 3-Aminopropyl triethoxysilane (HiMedia) then fixed in 20% acetone and embedded in paraffin. After that tissue sections were treated with IL-6 (Cell Signaling Technology, CST, #12153, United States), TNF-α (CST, #8242), and IL-10 (CST, #12163) for immunohistochemistry analysis was performed according to the manufacturer’s recommendations. Tissue sections were deparaffinized in xylene and gradual rehydration with ethanol (100–60%), then incubated in 1% sodium citrate buffer (pH 6.5) in a microwave oven at the optimum temperature for 25 min. Tissue sections were incubated in 3% Hydrogen peroxide for 15 min and washed in PBS for 10 min. Then incubated in humidity conditions by adding a secondary anti-rabbit antibody (HP05, Bangalore Genei) for 8 h at 4°C and section slides were rinsed 3–4 times with PBS for 5 min. After washing with PBS, the tissue sections were stained with 3, 3′-Diaminobenzidine and counterstained using hematoxylin. The tissue sections were dehydrated in ascending grades of ethanol (60–100%) then cleared with xylene, mounted using DPX and observed using light microscopy (20×). Control and inflammation control tissue sections were processed along with IL-6, TNF-α, and IL-10 antibodies in independent experiments. Negative control tissue sections were stained with secondary antibody alone in this experiment (Arjumand et al., 2011; Levkovich et al., 2013).

### Extraction of Total RNA From Rat Paw Tissues

Rat paw tissues (50 mg) were homogenized using liquid nitrogen and 1 mL of TRIzol reagent (Invitrogen, United States) was added. After TRIzoL treatment, RNA was suspended in 50 μL Diethylpyrocarbonate. Purity of RNA was determined based on the 260/280 nm ratio. The residual DNA was removed by treating RNA with DNase I (Promega). 1μg of DNase treated RNA was transcribed to cDNA by reverse transcriptase (TakaRa, RR036A).

#### qRT-PCR

The qRT-PCR reaction was performed in Light Cycler instrumentation by relative quantification software with fluorescence detector. In PCR reaction, each cycle as follows; 10 μL reaction composed of 5 μL of 2× SYBR Green PCR master mix, 2.50 μL of cDNA (30 ng/μL for genes), 0.5 μL of primer at 10 μM and 1.50 μL of PCR water. PCR conditions briefly, initial denaturation temperature at 95°C for 5 min, amplification and quantification of PCR cycles as follows; 10 s at 955°C, 15 s annealing temperature and 15 s at 72°C with fluorescence, melt curve program at 60–955°C with a temperature rate of 0.15°C s^−1^, fluorescence and final step to 35°C. The sequence of the primers used in this study was described in Table 6. Gene expressions in relation to each RNA extractions was performed in triplicates, mean values were used for the data analysis (Duary et al., 2012).

#### Data Analysis of Relative Gene Expressions

Quantitative data analysis by qRT-PCR is based on the number of cycles required for amplification generated fluorescence to reach the specific Ct values. RT- PCR amplification efficiencies were determined for each set of primers with the slope of a linear regression (Pfaffl, 2001). Standard curves were obtained by plotting the log cDNA values against Ct values (Rasmussen, 2001). Total expression ratio of genes of interest tested for significance using a randomization test in the relative gene expression software in the application for group wise comparison and statistical analysis. Randomization test as the part of the REST software tool and it was used to assess the statistical significance of regulation of the genes after normalization to GAPDH reference gene. For up-regulation, the factor of regulation is equal to the given value in the randomization data output box, for downregulation the regulation factor is demonstrated as a reciprocal value. In randomization test, randomly observed values in various samples to the values in control, relative gene expression estimated for each group and effect of randomization in each group was assessed.

### Level of Lipid Peroxidation in Paw Tissues

Lipid peroxidation was assessed by Thiobarbituric acid reactive substances (TBARS) in inflammation induced rat paw tissues. Stock solution contained the equal volume of 15% Trichloroacetic acid in Hydrochloride, 1:2 ratio of test sample and stock reagent were mixed in a centrifuge tube and boiled for15 min. After that precipitates were removed by the centrifugation and absorbance was read at 532 nm against blank. Experimental values were denoted as μM of malondialdehyde formed per min per mg of protein (Yagi, 1987).

### Antioxidant Enzyme Assays of Paw Tissues

Superoxide dismutase: 100 μL of tissue homogenate was incubated with ethanol and chloroform in an incubator shaker for 15 min then centrifuged for 5 min. Collected supernatant was mixed with 0.1 M Tris-hydrochloride, sterile water and 1 mM pyrogallol in 0.05 M Tris-hydrochloride, optical density (OD) at 420 nm was measured at 0, 1, and 2 min. Control samples containing 0.5 mL of distilled water treated against the blank and enzyme activity was represented as unit per mg protein (Marklund and Marklund, 1974).

Catalase: 100 μL of tissue homogenate was taken and 0.5 mL of 0.01 M PBS and 0.2 M Hydrogen peroxide were added and incubated for 10 min at room temperature. After incubation, the reaction was stopped by 1.0 mL of 5% dichromate acetic acid (1:3) and hydrogen peroxide in the range of 0–20 μM was treated. The sample were heated 10 min and the established green color measured at 570 nm. Catalase enzyme activity represented as μM of Hydrogen peroxide consumed per min per mg of protein (Satish Kumar et al., 2012).

Glutathione S-transferase: Assay mixture was composed of 0.1 M phosphate buffer, 1 mM of 1-Chloro- 2, 4-dinitrobenzene, 1 mM EDTA and 1 mM of glutathione. Reaction was initiated by addition of test samples and absorbance was read at 345 nm. Glutathione S-transferase enzyme activity was expressed as μM of 1-Chloro- 2, 4-dinitrobenzene utilized per min per mg of protein (Satish Kumar et al., 2012).

### *In vitro* DPPH Antioxidant Assay of Probiotic Strains

The antioxidant properties of *L. mucosae* AN1 and *L. fermentum* SNR1 on DPPH radical scavenging were performed. Reaction mixture consists 2 mL of 2, 2-diphenyl-1-picrylhydrazyl (DPPH, HiMedia) (0.2 mM in ethanol) and 2 mL of AN1 and SNR1 strains independently (10^8^ CFU/mL). The reaction mixture was incubated at 37°C for 30 min and scavenged DPPH was observed by measuring the decrease in OD at 517 nm. Ascorbic acid used as a standard drug and scavenging activity of bacterial strains (AN1 and SNR1) were determined using the following formula.

$$\begin{array}{lll} \left(\%\right) & = & [1-\text{sample }(\text{OD})/\text{blank }(\text{OD})]\times 100\\ & & (\mathrm{Amaretti et al.,}\;2013). \end{array}$$

### *In vitro* ABTS Antioxidant Assay

Antioxidant activity was determined using ABTS scavenging activity. Briefly, 7 mM of ABTS was dissolved in sterile distilled water, ABTS radical cations were produced by reacting with potassium persulfate then incubated in dark at 37°C for 15 h. After incubation time, ABTS radical cation solution was diluted with PBS and set OD of 0.70 at 734 nm. Two milliliter ABTS^+^ solution was added with 500 μL of *L. mucosae* AN1, *L. fermentum* SNR1 strains independently (10^8^ CFU/mL), incubated for 5 min and the measured absorbance at 734 nm. Three independent experiments were carried out and the percentage inhibition of absorbance was calculated using ascorbic acid standard curve (Ji et al., 2015).

### Data Analysis

All the statistical analysis were performed with the Origin and Microsoft Excel software, results were represented as mean ± standard error mean and analysis of the data was performed by ANOVA and Tukey’s multiple comparison.

## Results

### Carrageenan Induced Acute Inflammation

Carrageenan solution was injected into hind paw induced a progressive edema. Group I rats initial paw thickness found was 3.82 ± 0.13 mm, after 24 h, it was increased to 4.82 ± 0.10 mm. Group II inflammation control animals exhibited an increase in paw thickness at each interval of time. At *t* = 0 h the rat paw edema was 6.22 ± 0.10 mm and it was increased to 6.70 ± 0.62 mm at *t* = 1 h. After 24 h thickness of the rat paw was found to be decreased to 5.25 ± 0.04 mm. Group III animals were treated with Diclofenac sodium reference drug and rat paw thickness was found to be 5.02 ± 0.10, 6.40 ± 0.04, 6.25 ± 0.08, 5.03 ± 0.08 at 0, 1, 2, 4 h, respectively, and after 24 h the thickness of the rat paw was observed as5.06 ± 0.05 mm. In probiotic treated Group IV, paw thickness of the rats was found to be 5.60 ± 0.18 mm at 0 h and after 1 h, it was found to be increased to 6.20 ± 0.20 mm. After 2, 4, and 24 h of time intervals the thickness of rat paw was found to be decreased to 6.32 ± 0.60, 6.50 ± 0.22, 6.0 ± 0.30 mm, respectively. Group V rats paw edema was measured at 0 h was 4.70 ± 0.04 mm, which revealed an increase of 5.45 ± 0.34 mm at the end of 1 h. After 2, 4, and 24 h, the thickness of paw was decreased to 4.50 ± 0.07, 4.22 ± 0.20 and 5.20 ± 0.50 mm, respectively. In Group VI animals, at 0 h paw thickness of rats were found to be 5.20 ± 0.13 mm and after 1, 2, 4, and 24 h paw edema were recorded such as 5.30 ± 0.11, 5.82 ± 0.05, 5.85 ± 0.20, and 5.88 ± 0.65 mm, respectively.

In group VII rats, paw thickness at 0 h was 4.56 ± 0.071 mm which exhibited an increased paw volume at 1 h was 4.37 ± 0.070 mm. After 2, 4, and 24 h paw thickness was found to be 4.48 ± 0.03, 4.65 ± 0.0, and 4.92 ± 0.04 mm, respectively. Combination of encapsulated strains *L. mucosae* AN1 and *L. fermentum* SNR1 administered group VIII, the thickness of rat paw was found to be 4.60 ± 0.20 mm at 0 h, after 1 h it was increased to 4.95 ± 0.30 mm. At 2, 4, and 24 h the thickness of rat paw was increased to 5.08 ± 0.20, 5.48 ± 0.22, and 5.45 ± 0.01 mm, respectively.

**FIGURE 1:**
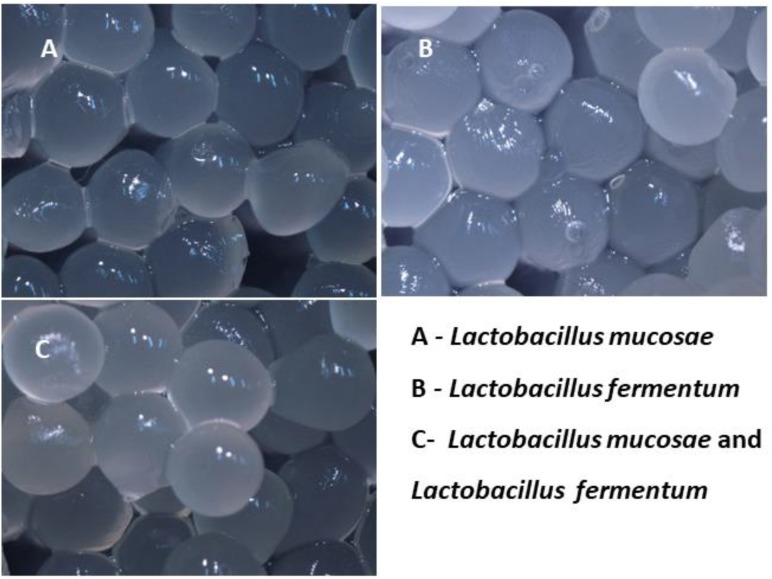
Microencapsulation of *L. mucosae* AN1 and *L. fermentum* SNR1.

**FIGURE 2:**
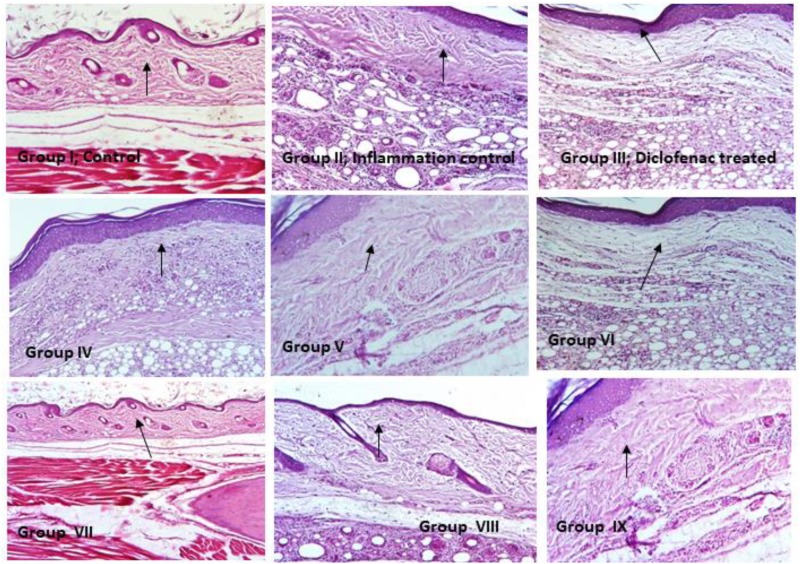
Hematoxylin and Eosin staining of rat paw tissue sections.

Similarly in group IX, which was administered with unencapsulated probiotic strains and at 0 h paw thickness of rats was recorded as 4.25 ± 0.10 mm. At 1 h time period, paw thickness was 4.38 ± 0.12 mm and after 24 h of duration, paw edema increased to 5.02 ± 0.55 mm. Experimental values were measured for six rats from each group (Figures 1, 2 and Table 2).

**Table 2:** Effects of probiotic strains *L. mucosae* AN1 and *L. fermentum* SNR1 on paw thickness of Wistar rats.

Groups	Before Inj.	(After inj.) 0 h	1 h	2 h	4 h	24 h	% Inhibition of edema at 24 h
I	3.80 ± 0.12	3.82 ± 0.13	3.84 ± 0.13	3.89 ± 0.13	4.04 ± 0.13	4.82 ± 0.10	–
II	4.05 ± 0.83	6.22 ± 0.10	6.70 ± 0.62	6.89 ± 0.10	5.50 ± 0.15	5.25 ± 0.04	–
III	3.40 ± 0.05	5.02 ± 0.10	6.40 ± 0.04	6.25 ± 0.08	5.03 ± 0.08	5.06 ± 0.05	40.02 ± 36
IV	5.00 ± 0.50	5.60 ± 0.18	6.20 ± 0.20	6.32 ± 0.60	6.50 ± 0.22	6.0 ± 0.30	40 ± 08
V	3.42 ± 0.73	4.70 ± 0.04	5.45 ± 0.34	4.50 ± 0.07	4.22 ± 0.20	5.20 ± 0.50	50 ± 16
VI	4.50 ± 0.10	5.20 ± 0.13	5.30 ± 0.11	5.82 ± 0.05	5.85 ± 0.20	5.88 ± 0.65	68 ± 28
VII	3.83 ± 0.12	4.56 ± 0.071	4.37 ± 0.070	4.48 ± 0.03	4.65 ± 0.01	4.92 ± 0.04	36 ± 10
VIII	3.78 ± 0.13	4.60 ± 0.20	4.95 ± 0.30	5.08 ± 0.20	5.48 ± 0.22	5.45 ± 0.01	85 ± 13
IX	4.02 ± 0.01	4.25 ± 0.10	4.38 ± 0.12	4.85 ± 0.15	5.35 ± 0.04	5.02 ± 0.55	77 ± 25

*Data represented as mean paw thickness (mm) ± SEM, n = 6.*

In results, paw thickness was observed that encapsulated *L. mucosae* AN1 and *L. fermentum* SNR1 administered group VIII exhibited 85 ± 13% of paw edema volume inhibition and the unencapsulated combination of the strains treated, group IX exhibited 77 ± 25% of inhibition of paw edema was determined. Diclofenac sodium treated group III revealed 40.02 ± 36% of edema reduction was observed in carrageenan-induced acute inflammation in Wistar male rats.

### Hematoxylin and Eosin Staining

Inflammation induced by carrageenan experiments were performed and observed to be rat paw was associated with cellular infiltration, edema and granuloma formation. In inflammation control (Group II), revealed significantly increased in inflammatory leukocyte infiltration than the control rats (Group I). Necrotic and degenerative changes were found in control group (Group I) and inflammation control (Group II) rats, while in histopathological analysis of probiotic strains *L. mucosae* AN1 and *L. fermentum* SNR1 administered groups (Groups IV–IX) exhibited moderate destruction and less degenerative changes in the morphology of rat paw tissue sections (Figure 2).

### Hematological Parameters

In inflammation control (Group II), there was found increased levels of ESR and C-reactive protein levels were observed than probiotic administered groups (Groups IV–IX). RBC, hemoglobin, WBC and platelet counts were found to be normal in all the groups (Table 3).

**Table 3:** Hematological parameters.

Groups	I	II	III	IV	V	VI	VII	VIII	IX
Hb (g/dL)	10.0 ± 0.12	8.92 ± 0.36	11.00 ± 0.50	8.60 ± 0.55	12.50 ± 0.44	7.00 ± 0.25	10.80 ± 0.32	10.00 ± 0.86	10.80 ± 0.7
WBC	7900 ± 0.16	7800 ± 0.08	8300 ± 0.02	7500 ± 0.53	7300 ± 0.62	7100 ± 0.90	10,000 ± 0.6	13,300 ± 0.11	8800 ± 0.65
RBC	4.60 ± 0.72	3.8 ± 0.81	5.17 ± 0.05	1.68 ± 0.40	6.08 ± 0.18	2.94 ± 0.75	5.16 ± 0.20	4.26 ± 0.66	4.52 ± 0.42
Platelets	7 × 10^3^	8 × 10^3^	6.77 × 10^3^	14 × 10^3^	12 × 10^3^	6 × 10^3^	8 × 10^3^	10 × 10^3^	13 × 10^3^
ESR (mm/h)	1.80 ± 0.1	3.7 ± 0.2	1.53 ± 0.5	1.40 ± 1.20	2.10 ± 1.04	2.22 ± 0.15	2.10 ± 0.5	1.11 ± 0.8	2.41 ± 1.50
CRP (mg/dL)	4.20 ± 0.6	6.0 ± 1.01	4.11 ± 0.08	4.22 ± 1.10	4.25 ± 0.15	4.20 ± 0.31	4.20 ± 1.28	4.00 ± 0.50	4.32 ± 0.10

*Data represented as mean ± SEM, n = 6; RBC and WBC cell counts were represented as million cells/column.*

### Cytokines Assay

In rat serum, proinflammatory cytokines levels such as interleukin 6 and TNF-*α* were high in inflammation control (Group II) was found to be 53.01 ± 0.105 and 525.00 ± 0.220 pg/mL, respectively, and 26.016 ± 0.025 pg/mL of IL-10 levels were determined. In diclofenac sodium treated (Group III) rats interleukin IL6, TNF*α*, and IL-10 levels were determined to be 66.10 ± 0.120, 475.02 ± 0.162, and 32.265 ± 0.030 pg/mL, respectively. On the contrary, *L. mucosae* AN1 and *L. fermentum* SNR1 administered groups were exhibited anti-inflammatory properties, particularly in group VIII which was treated with the combination of encapsulated AN1 and SNR1 strains. Interleukin (cytokines) levels such as IL6, TNF*α*, and IL10 in group VIII rats serum was determined to be 47.15 ± 0.120, 431.01 ± 0.720, 42.005 ± 0.580 pg/mL, respectively (Table 4).

**Table 4:** Effect of *L. mucosae* AN1 and *L. fermentum* SNR1 on cytokines expression.

Groups	Groups composition	IL-6 (pg/mL)	TNF-α (pg/mL)	IL-10 (pg/mL)
I	Control	68.25 ± 0.302	672.15 ± 0.135	16.05 ± 0.005
II	Inflammation Control	53.01 ± 0.105	525.00 ± 0.220^a^	26.016 ± 0.025
III	Diclofenac treated	66.10 ± 0.120^a^	475.02 ± 0.162	32.265 ± 0.030
IV	*Lactobacillus mucosae*	42.60 ± 0.903	432.00 ± 0.320	37.05 ± 0.239
V	*L. mucosae*	43.00 ± 0.105	430.09 ± 0.143^a^	36.05 ± 0.320
VI	*Lactobacillus fermentum*	46.50 ± 0.301	420.32 ± 0.105^a^	40.125 ± 0.410
VII	*L. fermentum*	46.93 ± 0.549^a^	425.90 ± 0.783	40.095 ± 0.154
VIII	*L. mucosae* and *L. fermentum*	47.15 ± 0.120	431.01 ± 0.720^a^	42.005 ± 0.580
IX	*L. mucosae* and *L. fermentum*	44.00 ± 0.320^a^	425.00 ± 0.403	41.005 ± 0.240^a^

*Data represented as mean ± SEM of six rats per group, experimental values along the columns are statistically significant at P < 0.05 when compared with control.*

### Immunohistochemistry

**FIGURE 3:**
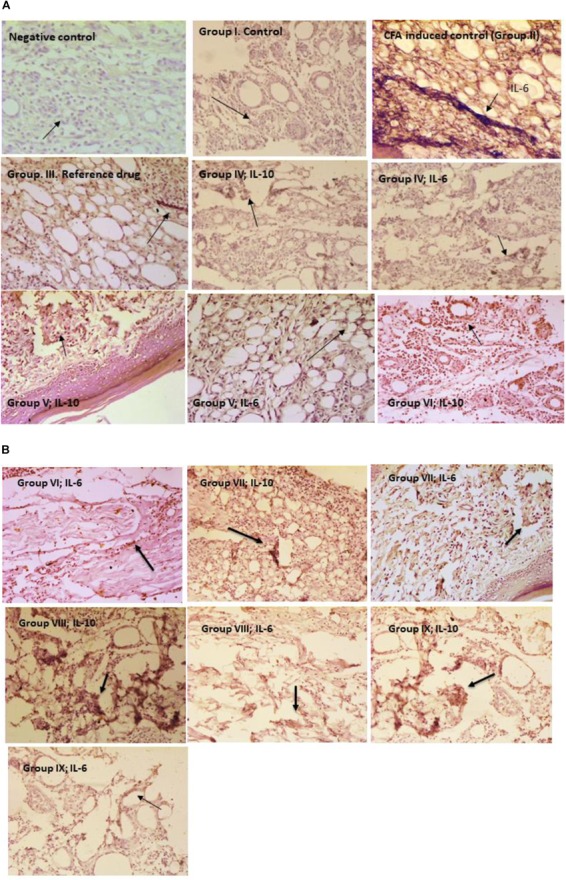
**(A)** Immunohistochemistry of rat paw tissues. **(B)** Immunohistochemistry of rat paw tissues.

Immunohistochemical evaluation of microencapsulated and unencapsulated probiotic strains administered groups were assessed for the interleukins expression of IL-6 and IL-10 in paw tissues. Inflammation control (Group II) rats paw tissue sections were revealed high expression of IL-6 than IL-10 in staining. Microencapsulated probiotic strains administered group (VIII) was revealed high expressions of IL-10 (an anti-inflammatory cytokine) and low expressions of IL-6 (a proinflammatory cytokine) were observed in the immunohistochemical staining. In Group XI, interleukins IL-10 and IL-6 expression levels were observed to be low when compared to the Group VIII paw tissue sections (Figures 3A,B).

### qRT-PCR

**FIGURE 4:**
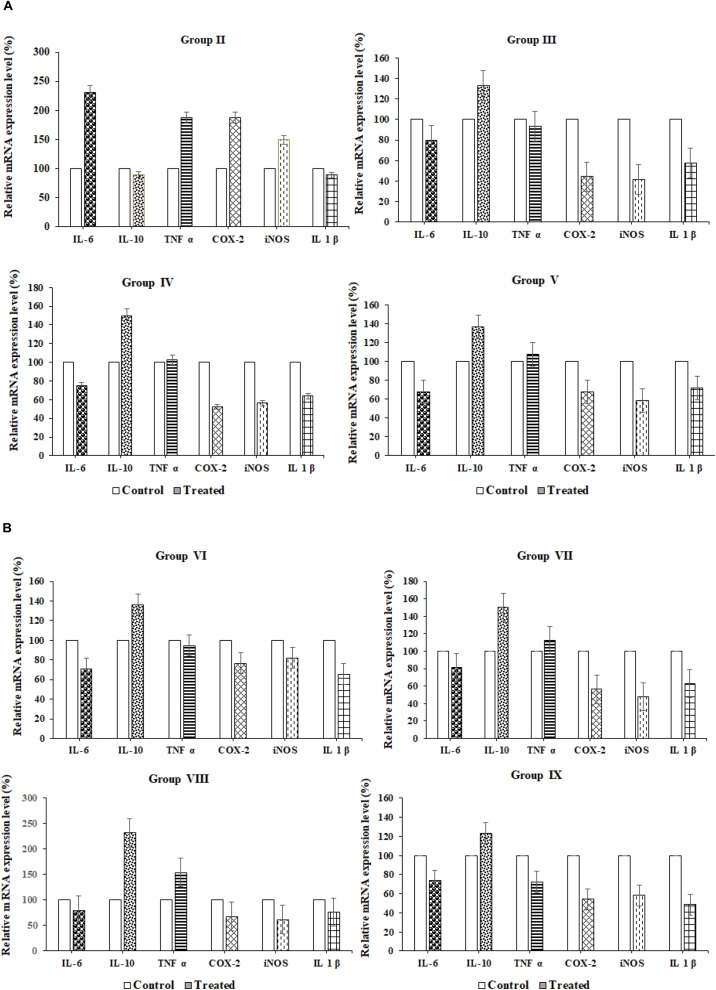
**(A)** qRT-PCR. **(B)** qRT-PCR.

Relative proinflammatory cytokine gene expression (IL6, TNF *α*, COX2, iNOS, and IL1β) and anti-inflammatory (IL10) cytokines gene expressions in the paw tissues of probiotic strains administered groups were analyzed using reverse transcription quantitative PCR (qRT-PCR). Results were observed that in the normal control group I proinflammatory cytokine genes were upregulated and anti-inflammatory (IL10) genes were downregulated. In inflammation control (Group II) cytokine genes such as IL-6, TNF- *α*, COX-2, iNOS, and IL-1β were upregulated and relative mRNA expression was found to be 75, 92, 40, 35, and 53%, respectively, whereas anti-inflammatory cytokine (IL-10) was down-regulated and expression of IL-10levels found to be 135%. The diclofenac sodium reference drug-treated Group III rats were exhibited up-regulation of anti-inflammatory cytokine (IL-10, 135%) and down-regulation of proinflammatory cytokines such as IL-6, TNF- *α*, COX-2, iNOS, and IL-1β; gene expression levels 78, 90, 40, 38, 60% were observed, respectively. In group VIII, revealed 240% of relative mRNA expression of IL-10 was recorded and down-regulation of proinflammatory cytokines such as IL-6, TNF- *α*, COX-2, iNOS, and IL-1β and their relative mRNA expressions were observed78, 140, 55, 44, and 56% respectively. Among all the probiotic administered animal groups, VIII and IX groups have exhibited better anti-inflammatory properties than the remaining Wistar rat groups (IV, V, VI, and VII) (Table 6 and Figures 4A,B).

### Level of Lipid Peroxidation and Antioxidant Enzyme Assays

Rat in groups I, II and III were analyzed for the formation of TBARS (TBARS-Thiobarbituric acid reactive substances) such as 2.00 ± 0.127, 2.17 ± 0.080, and 0.104 ± 0.02 per min per mg of protein were observed, respectively. In probiotic administered groups, particularly in group VIII, lipid peroxides formation was found to be 0.40 ± 0.02 min^−1^ mg of protein^−1^. Probiotic bacteria administered groups such as group IV, V, VI, VII, and IX were exhibited least TBARS formation of 1.50 ± 0.72, 0.092 ± 0.54, 0.62 ± 0.18, 0.65 ± 0.12, and 0.74 ± 0.22 min^−1^ mg of protein^−1^, respectively, in the inflammation induced paw tissue homogenates (Table 5).

**Table 5:** Level of lipid peroxidation and antioxidant enzyme assays.

Groups	Lipid peroxides	SOD	GST	CAT
I	2.00 ± 0.127^a^	1.890 ± 0.07^a^	0.42 ± 0.052^a^	0.625 ± 0.011^a^
II	2.17 ± 0.080^b^	1.54 ± 0.42^b^	0.68 ± 0.029^b^	0.832 ± 0.64^b^
III	0.104 ± 0.02^c^	1.24 ± 0.81^c^	2.12 ± 0.04^c^	1.5 ± 0.02^c^
IV	1.50 ± 0.72^d^	3.11 ± 0.15^d^	1.56 ± 0.226^d^	1.6 ± 0.084^d^
V	0.092 ± 0.54^e^	3.15 ± 0.617^e^	2.25 ± 0.08^e^	2.15 ± 0.52^e^
VI	0.62 ± 0.18^f^	2.84 ± 0.20^f^	0.91 ± 0.59^f^	1.20 ± 0.31^f^
VII	0.65 ± 0.12^g^	3.15 ± 0.65^g^	0.80 ± 0.11^g^	1.04 ± 0.05^g^
VIII	0.40 ± 0.02^h^	3.02 ± 0.35^h^	2.22 ± 0.63^h^	2.24 ± 0.07^h^
IX	0.74 ± 0.22^i^	2.08 ± 0.07^i^	1.02 ± 0.30^i^	1.32 ± 0.068^i^

*Groups were treated as described in the Materials and Methods section. Values are represented as mean ± standard deviation from six rats in each group and values not indicated a common superscript letter (a–i) differ significantly at P < 0.05. Experimental values are expressed as follows: Lipid peroxidation, TBARS formed min^−1^ mg of protein^−1^; SOD (Superoxide dismutase), units/mg of protein; GST (Glutathione-S-transferase), μM of 1-Chloro- 2, 4-dinitrobenzene utilized min^−1^ mg of protein^−1^; CAT (Catalase), μM H_2_O_2_ utilized min^−1^ mg of protein^−1^.*

**Table 6:** Sequences of the primers used in qRT-PCR.

Genes	Forward primers (5′–3′)	Reverse primers (5′–3′)	Annealing	Tm	Amplicon
			temp. (° C)	(° C)	size (bp)
TNF-α	ACGCTCTTCTGTCTACTG	GGATGAACACGCCAGTCG	54	52.68	212
IL-10	GAAGTGATGCCCCAGGCAGA	ACGTAGGCTTCTATGCAGT	54	58.88	253
IL-6	CATGTTCTCTGGGAAATC GTGG	AACGCACTAGGTTTGCCGA GTA	54	54.84	209
IL-1β	CCAGGATGAGGACCCAAG	TCCCGACCATTGCTGTTT	54	52.60	249
iNOS	CCCAGCCTCAAGTCTTATTTCCTC	GCACTCAGCAGCAAGTTCCATC	54	57.38	220
COX-2	CCTGTGCCTGATGATTGC	CTGATGCGTGAAGTGCTG	54	50.32	96
GAPDH	GCAAGTTCAACGGCACAGCACAG	GCCAGTAGACTCCACGACAT	54	58.84	124

Superoxide dismutase, Glutathione S-transferase and Catalase in rat paw tissues were assessed for antioxidant properties. Superoxide dismutase activity was determined as 1.890 ± 0.07 units per mg of protein and in the probiotic administered groups paw tissue homogenates such as IV, V, VII, and VIII were revealed the better activity of 3.11 ± 0.15, 3.15 ± 0.617, 3.15 ± 0.65, 3.02 ± 0.35 units/mg of protein, respectively. Glutathione S-transferase activity of tissues in rat groups such as group I, group II, and group III were determined to be 0.42 ± 0.052, 0.68 ± 0.029, 2.12 ± 0.04 μM of 1-Chloro- 2, 4-dinitrobenzene utilized per min per mg of protein, whereas in probiotic bacteria treated tissue homogenates of group V and VIII were found to be high activity of 2.25 ± 0.08, 2.22 ± 0.63 μM of 1-Chloro- 2, 4-dinitrobenzene utilized per min per mg of protein, respectively. Catalase activity of group I, II and III tissues was determined as 0.625 ± 0.011, 0.832 ± 0.64, 1.5 ± 0.02 μM H_2_O_2_ utilized per min per mg of protein, respectively. In probiotic bacteria administered groups which are exhibited high catalase activity in group V (Encapsulated *L. mucosae* AN1) and VIII (Encapsulated *L. mucosae* AN1 and *L. fermentum* SNR1) were determined to be 2.15 ± 0.52, 2.24 ± 0.07 μM H_2_O_2_ utilized per min per mg of protein, respectively.

### *In vitro* Antioxidant Properties of Probiotic Strains

Antioxidant properties of *L. mucosae* AN1 and *L. fermentum* SNR1 were exhibited maximum DPPH free radical scavenging ability was determined to be 66.53 ± 1.15, 67.25 ± 1.64%, respectively. Scavenging ability of the strains AN1 and SNR1 against ABTS radicals were analyzed and determined to be 75.00 ± 1.73 and 80.83 ± 1.81%, respectively. The probiotic strains have exhibited similar antioxidant activities when compared with ascorbic acid control in this study.

## Discussion

Objective of the present study was *in vivo* approach to investigate antioxidant, anti-inflammatory characteristics of *Lactobacillus mucosae* AN1, *Lactobacillus fermentum* SNR1 in Wistar albino rats. Probiotic bacteria modulates immunity by interacting with the indigenous gut microflora for immunomodulation (Kirjavainen et al., 1999; Johnson-Henry et al., 2004). Microencapsulated and oral probiotics *L. mucosae* AN1 and *L. fermentum* SNR1 administration results in protection against inflammation symptoms and the strains have exhibited therapeutic properties. The advantages of viable bacterial vaccines, they mimic natural infection and intrinsic adjuvant characteristics. Components of non-pathogenic food related microbes are evaluated as oral vaccines (Amdekar et al., 2010). Complete Freund’s adjuvant-induced chronic inflammation and carrageenan-induced acute edema approaches are suitable for the evaluation of anti-inflammatory properties in the animal model. These methods have been used frequently to assess the anti-edematous properties; carrageenan injection was used for the release of inflammation-causing metabolites such as leukotrienes, prostaglandins, bradykinin, histamine, and TNF-*α* (Solanki et al., 2015).

In the present study, *L. mucosae* AN1 and *L. fermentum* SNR1 exhibited a significant decrease in rat paw edema. Microencapsulated bacterial strains (Group VIII) and unencapsulated strains (Group IX) administered rats have exhibited better paw edema inhibition. Previous studies were also reported that sodium alginate microencapsulated *L. sporogenes* and *B. bifidum* strains inhibited carrageenan-induced paw edema (Solanki et al., 2015). After induction of inflammation in acute inflammation process, the release of histamines, serotonin, kinins and in the second phase, release of prostaglandins were observed (Brooks and Day, 1991). Anti-inflammatory properties of *L. fermentum* could release glutathione and it prevents colonic inflammation in colitis rats (Peran et al., 2006). *Lactobacillus casei* significantly decreased interleukins TNF-α, IL-6 and adhered to surface molecules thereby suppressing the signaling pathway of IL-6, TNF-α (Lee et al., 2008). Prostaglandins cause pain and inflammation, cyclooxygenase is the rate-limiting enzyme in synthesis of prostaglandins and regulates critical physiological mechanisms such as immune responses and in kidney functioning (Dubois et al., 1998). Lipoxygenase and cyclooxygenase pathways play an important role in the inflammation (Giuliano and Warner, 2002). *Lactobacillus casei* induced COX-2 inhibition by inhibiting the proinflammatory interleukins are of complex interactions involved in the inflammation (Amdekar et al., 2011).

In blood analysis, ESR and CRP levels were elevated in group I, II, and III animals, whereas in probiotic (*L. mucosae* AN1 and *L. fermentum* SNR1) administered groups (IV–IX) were revealed normal levels in all the parameters such as hemoglobin content, WBC, RBC, platelet counts, ESR and CRP levels. In hematoxylin and eosin staining results were revealed that the morphology of epidermis of inflamed tissues (Group II), paw edema was clearly observed. The probiotic administered groups have exhibited no inflammation in the epidermis of tissue sections (Group IV to IX).

The immunohistochemistry results clearly showed in group VIII, IL-10 expressions were high and IL-6 expressions were low in immunohistochemical staining. Similarly, in group IX which was treated with the combination of non-encapsulated *L. fermentum* SNR1 and *L. mucosae* AN1 also revealed similar results but in group VIII paw tissue staining was exhibited better expression of IL-10 than IL-6. In previous studies, administration of *L. rhamnosus* GG, *B. animalis*, and *Lactis* Bb12 in adult human clinical trials revealed anti-inflammatory properties (Kekkonen et al., 2008). In the humanized mouse model reported that *Lactobacillus rhamnosus* and *Propionibacterium freudenreichii* induced to downregulate the pro-inflammatory changes by a high-fat food (Oksaharju et al., 2013). IL-10 is an anti-inflammatory cytokine that plays major role in host immunity against pathogenic invaders and preventing autoimmune and inflammatory diseases (Sabat et al., 2010).

In cytokines assay and qRT-PCR results, the probiotic bacteria *L. fermentum* SNR1, *L. mucosae* AN1 strains exhibited anti-inflammatory cytokines upregulation and proinflammatory cytokines downregulation were observed. In previous reports, oral administration of *Lactobacillus* bacteria exhibited anti-inflammatory properties (Oksaharju et al., 2013). IL-10 was found to be a potent macrophage deactivator and it blocked proinflammatory cytokines in human monocytes (Trushin et al., 2003). Prostaglandins synthesis downregulated by IL-4 and IL-10 and downregulated COX-2 synthesis (Oksaharju et al., 2013). *L. casei* inactivates NF-κB synthesis and induction of TNF-α upregulated by the NF-κB which in turn responsible for COX-2 synthesis (Peña and Versalovic, 2003). VSL#3 probiotic administration reduced the level of hepatic IL-6, but no effect on the hepatic TNF-α levels in a sepsis mouse model (Ewaschuk et al., 2007). Immune mediated mechanisms in the metabolic scenario have increased evidencing the role and several probiotic bacteria are able to modulate immune system by stimulating the release of different cytokines (Kwon et al., 2010). *Lactobacillus jensenii* TL2937 modulates intestinal TLR4-inflammatory signaling pathways for improving human and animal health (Villena and Kitazawa, 2014).

In antioxidant evaluation, probiotic strains administered (Group IV–IX) rats rat paw tissues of lipid peroxides formation was very low levels whereas in inflammation control (Group II) was reported high in lipid peroxide formation. Antioxidant enzyme activities of probiotic administered rat paw tissues were observed high when compared to control tissue homogenates. The probiotic strains administered groups were observed that there were significant in reduced levels of lipid peroxides formation and increased antioxidant enzyme activities when compared to control and diclofenac sodium drug-treated rats. In *in vitro* evaluation of the antioxidant properties using DPPH and ABTS radical scavenging assays, *L. mucosae* AN1 and *L. fermentum* SNR1 have revealed good free radical inhibition and reduction in ABTS cation radicals. Previously *L. casei* exhibited antioxidant abilities on hyperlipidemic rats through the improvement of malondialdehyde levels and antioxidant properties (Zhang et al., 2010). *L. gasseri* producing superoxide dismutase enzyme has a potent anti-inflammatory activity and that decreased the severity of colitis in mouse model (Carroll et al., 2007; Mileti et al., 2009).

In summary, the probiotic strains (*L. mucosae* AN1 and *L. fermentum* SNR1) were significantly decreased the paw edema inflammation and in the hematoxylin-eosin staining, the morphology of the probiotic administered groups were exhibited effective anti-inflammatory properties. Immunohistochemistry staining results revealed that anti-inflammatory cytokine IL-10 expressions were high when compared to proinflammatory cytokine IL-6 in the rat paw tissue sections. qRT-PCR and ELISA results were revealed that there is upregulation of anti-inflammatory cytokine and down-regulation of proinflammatory cytokines. Therefore the probable mechanism in antioxidant probiotic bacteria administered Wistar albino rats could be inhibition of prostaglandins synthesis due to high expression of anti-inflammatory cytokines. Hence, *L. mucosae* AN1 and *L. fermentum* SNR1 revealed antioxidant and anti-inflammatory properties they may be used in the animal model for their beneficial health.

## Author Contributions

RA designed this research work, executed the experiments, interpreted the results and wrote the manuscript. DA provided technical assistance during the animal study. All the authors have contributed equally and approved this manuscript.

## Conflict of Interest Statement

The authors declare that the research was conducted in the absence of any commercial or financial relationships that could be construed as a potential conflict of interest.
